# Online Model Updating and Dynamic Learning Rate-Based Robust Object Tracking

**DOI:** 10.3390/s18072046

**Published:** 2018-06-26

**Authors:** Md Mojahidul Islam, Guoqing Hu, Qianbo Liu

**Affiliations:** 1 School of Mechanical and Automotive Engineering, South China University of Technology, Guangzhou 510641, China; mdmojahidul.islam@yahoo.com (M.M.I.); hhylqb@sina.com (Q.L.); 2 Department of Computer Science and Engineering, Islamic University, Kushtia 7003, Bangladesh

**Keywords:** object tracking, machine learning, correlation filter, occlusion detection, scale adaptation, online model updating, dynamic learning rate

## Abstract

Robust visual tracking is a significant and challenging issue in computer vision-related research fields and has attracted an immense amount of attention from researchers. Due to various practical applications, many studies have been done that have introduced numerous algorithms. It is considered to be a challenging problem due to the unpredictability of various real-time situations, such as illumination variations, occlusion, fast motion, deformation, and scale variation, even though we only know the initial target position. To address these matters, we used a kernelized-correlation-filter-based translation filter with the integration of multiple features such as histogram of oriented gradients (HOG) and color attributes. These powerful features are useful to differentiate the target from the surrounding background and are effective for motion blur and illumination variations. To minimize the scale variation problem, we designed a correlation-filter-based scale filter. The proposed adaptive model’s updating and dynamic learning rate strategies based on a peak-to-sidelobe ratio effectively reduce model-drifting problems by avoiding noisy appearance changes. The experiment results show that our method provides the best performance compared to other methods, with a distance precision score of 79.9%, overlap success score of 59.0%, and an average running speed of 74 frames per second on the object tracking benchmark (OTB-2015).

## 1. Introduction

Robust visual object tracking has been an effective research subject in computer vision in the current decades. Techniques for visual object tracking are not only involved in practical applications, including face tracking [1,2], traffic analysis [3,4], and human–computer interaction [5,6], but are also applied to different video processing methods. The common strategy of visual tracking is to identify the target object by a bounding box over consecutive frames, where an initial target position is set in the start frame. Due to the practical applications of visual tracking, many research works have been performed and different strategies have been proposed [1,2,3,4,5,6]. Although single-object tracking is a well-researched topic and has achieved great improvements over the past decades, it remains a challenging problem to design efficient and robust trackers that can handle all challenging situations, such as deformation, abrupt motion change, partial occlusion, full occlusion, cluttered backgrounds, illumination changes, and large variations in the viewpoint and pose of the target.

Most of the object tracking techniques are categorized into two approaches—generative and discriminative. Generative tracking methods train the target object without considering the surrounding background information. This approach can control the partial missing data that occurs when the target is partially occluded. Eigentracking [7], incremental visual tracking [8], and circulant sparse tracking [9] are some generative-tracking-based algorithms. Contrary to the generative approach, the discriminative approach uses a binary classifier to differentiate the target from it surrounding background in the consecutive frames. Some examples of discriminative-tracking-based algorithms are ensemble tracking [10], discriminative tracking features [11], structured output SVMs [12], etc. These approaches are also known as tracking by detection and provide excellent results in visual tracking. Besides the single-object tracking algorithms, multiobject tracking algorithms [13,14] also use the tracking-by-detection principle. In [13], a multiperson tracking algorithm was proposed based on a dynamic appearance model. This study introduced a new appearance-modeling approach that provides exact appearance affinities to guide data association. In [14], novel relational appearance features and motion-patterns-learning-based data association techniques were proposed for multiobject tracking. Recently, correlation filters with handcraft features [15,16,17,18,19,20] and deep features-based correlation filters [21,22] have been used for efficient and effective visual tracking. Some trackers introduce part-based information into the correlation filter framework [23,24,25]. These trackers provide an evident component that integrates the part information in the training. Recently, Li et al. [26] proposed scene-aware adaptive-updating-based visual tracking using correlation filters (AKCF). The AKCF algorithm uses a different learning rate for model updates based on the scene classification, such as deformation, partial occlusion, long-term occlusion, and other scenes. In the convolution theorem [27], the convolution between two functions can be calculated in the frequency domain as an element-wise product. Thus, all the operations in the correlation filter can be performed in the Fourier domain to find the similarity between an input and the trained filter. These characteristics of correlation filters increase the computational speed at hundreds of frames per second.

However, among the existing correlation-filter-based methods, such as the circulant structure tracker with kernel trick (CSK) [16], the kernelized correlation filter (KCF) [18], color tracking (ACT) [19], scale-adaptive multifeature integration (SAMF) [20], discriminate scale space tracking (DSST) [17], correlation-filter-based tracking with a Siamese network (CFNet) [21], and hierarchical convolutional features (HCF) [22], have some limitations. The above trackers update the learned filter with a fixed learning rate using the moving average strategy to cope with recent appearance changes in consecutive image sequences. Since such model-updating procedures are suitable for short-term appearance variations, model-drifting problem occurs due to the noisy model updates in the subsequent frames and cannot recover the target from long-term occlusion and fast-motion variations. Another problem is scale variation, since these methods cannot adjust large-scale variations efficiently.

To address the above issues, we first constructed a translation filter using a kernel correlation filter to determine the target translation. To improve the accuracy, we integrated histogram of oriented gradients (HOG), color naming, and gray intensity features that are efficient to describe the target object model. Our proposed object search area size is effective to put the target object inside the search window at the time of large occlusion and abrupt motion change. We integrated the peak-to-sidelobe ratio (PSR) strategy in our method to detect appearance changes such as occlusion. The online model updating technique based on the PSR value reduces the model-drifting problem significantly.

To the best of our knowledge, we are the first to integrate some techniques, including KCF [18], multiple features [19,28], scale estimation [17], and peak-to-sidelobe ratio (PSR) [15], into a single tracking framework. We also introduced a dynamic learning-rate-based online model updating technique. Our tracking method is simple but effective in visual object tracking tasks in terms of tracking accuracy, robustness, and speed.

The principal contributions of this study are as follows: First, we study a new characteristic of the PSR values that are used to detect appearance changes and restrict the model updating scheme with no extra computational burden. This is important for the real-time performance of the tracking algorithm. Second, we propose a novel dynamic model learning rate using the current frame PSR score and others historical PSR scores. Third, we integrate multiple features such as HOG, color naming, and gray intensity features to boost the tracking performance. Fourth, numerous experiments have been carried out on the three large and challenging benchmark datasets, namely, Object Tracking Benchmark (OTB-2015) [29], Temple Color 128 [30], and MEEM [31], with several evaluation metrics, and the experiment outputs demonstrated that our method is highly efficient, robust, and runs at a speed of 74 frames per second.

## 2. Related Work

Online visual object tracking has been a popular research topic in computer vision and has been widely discuss in the literature [32,33]. The results comparison and evaluation methodology were discussed in [29,30]. In this part, we mainly introduce some tracking methods that are closely related in our present work.

In recent years, correlation-filter-based tracking techniques have shown impressive performance on the benchmark datasets [29,30]. Bolme et al. [15] proposed the first correlation-filter-based minimum output sum of squared error (MOSSE) tracker, which used the convolution theorem and a single-channel feature to accelerate the tracking speed. Henriques et al. [16] proposed a circulant structure tracker with a kernel trick (CSK), which introduced a circulant structure patch to improve the classifier by increasing the negative samples and integrating the kernel strategy with the correlation filter. Henriques et al. [18] improved the CSK tracker and proposed a high-speed kernelized correlation filter by integrating the kernel method into ridge regression and HOG features to enhance the overall tracking outputs. Danelljan et al. [19] proposed an adaptive color naming visual tracker (CN) to improve the CSK method by introducing a powerful color attributes feature for colored objects and a feature dimension reduction technique to increase the computational speed in the object tracking task. Valmadre et al. [21] introduced the CFNet, which attempts to increase the tracking speed without a tracking-accuracy drop by using correlation filters and low-level convolutional neural network (CNN) features. Ma et al. [22] introduced hierarchical convolutional features (HCF) to learn multiple kernel correlation filters for object tracking. Lukežič et al. [34] introduced a discriminative correlation filter based on channel reliability and spatial reliability strategies that help to enlarge the search area and increase the tracking of nonrectangular objects. Danelljan et al. [35] introduced a new method for training continuous convolution filters and integrating multiresolution deep features. This algorithm provides high accuracy, but the tracking speed is considerably low due to the high computational cost.

The abovementioned correlation trackers [15,16,18,19,21,22] use a fixed bounding box size over the tracking and concentrate to find the target position by estimating target translation. This restriction runs into problems when encountering target scale variations. Li and Zhu [20] first introduced a scale-adaptive multifeature correlation filter tracker (SAMF) to address this issue. However, this method has higher computational costs because the translation filter has to be applied at different resolutions to determine the accurate target size. Danelljan et al. [17] proposed a separate two-dimensional translation filter to estimate the target translation and a separate one-dimensional scale filter for target scale estimation that provide better scale results than SAMF [20]. To increase the frame rate along with robustness, Danelljan et al. [36] extended DSST with a feature dimension reduction technique and extended the search area of the target object without reducing the real-time performance. These three trackers extract target information on different scales to estimate the accurate target size. However, these methods are limited in predicting the diversity of target appearance, occlusion, and fast-object movement. To address the long-term tracking, Ma et al. [37] proposed long-term tracking with a random fern classifier and a redetection module to detect the target when tracking failure occurs. Hong et al. [38] integrated cognitive psychology principles to adapt the target appearance changes using short- and long-term memory. To handle occlusion and object deformation, some trackers divide the target objects into different parts [23,24,25]. Liu et al. [23] introduced an efficient method to measure the performance of several parts and integrate the correlation outputs of different parts. Lukežič et al. [24] proposed layered part based correlation filter trackers that use a geometrically constrained constellation of the local correlation filters to locate the target object. The reliable patch tracker (RPT) [25] identifies the reliable patches from the input image and exploits motion trajectories to differentiate them from the background.

Our studies are inspired by KCF, DSST, and long-term tracking strategies. We improve well-known kernelized correlation filter methods with the integration of multiple features, scale estimation, selective model updating, and online model learning-rate strategies. Because of the processing speed of correlation tracking, occlusion handling based on PSR, accurate scale change estimations, and an online adaptive learning rate, our method effectively handles occlusions, scale variations, and appearance model-drifting problems.

## 3. Methodology

**Figure 1:**
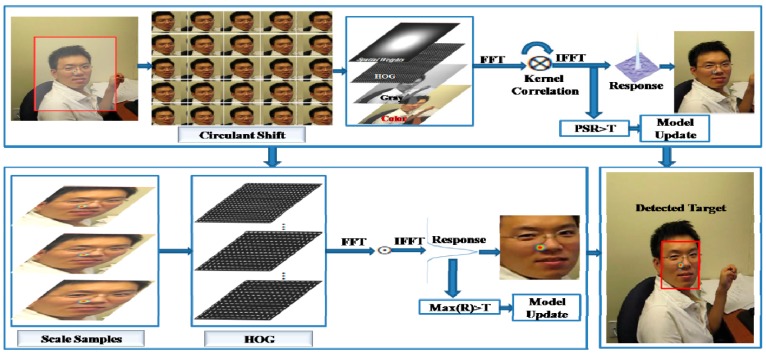
Block diagram of our proposed method.

In this part, we describe the baseline tracker and our proposed method. Algorithm 1 presents the outline of our method and the flow diagram of our method is shown in Figure 1. Our tracking strategies include a KCF tracker, multifeature integration, scale variation estimation, online model updating, and dynamic learning-rate adaptation.
**Algorithm 1.** Proposed tracking algorithm.**Require:** An input image sequence and object target locations $P(x_{1},y_{1},w_{1},h_{1})$ in the first frame. **Ensure:** Target object locations in the subsequent frames $P(x_{t},y_{t},w_{t},h_{t}),$ where t=2,3,…  **repeat**
//Translation calculationCalculate the correlation response map $R_{z}$ using (6) and estimate the target object location $\left(x_{t},y_{t}\right)$ by finding the maximum response position from $R_{z}$;//Scale estimationConstruct target feature pyramid around the position $\left(x_{t},y_{t}\right)$ and extract the HOGfeatures after resizing the image patch with same size and calculate the scale correlation response map $R_{s}$ using (6);Determine the optimal scale s using (12);Calculate the PSR score using (8);Compute dynamic learning rate $n_{t}$ using (9) and (10);//Translation correlation model updateif $PSR>T_{1}$ thenUpdate the translation model parameters using (11);End//Scale correlation model updateif $\max(R_{s})>T_{2}$ thenUpdate the scale model parameters using (13);End Until end of the image sequences;

### 3.1. KCF Tracker

We built our proposed method based on the KCF [18] method because it is simple, fast, and it provides high performance while considering limited training samples and the necessary computation in the training and detection steps using Fast Fourier Transform (FFT). The KCF tracker used a circulant structure matrix and learned a classifier of the target from an input image. The classifier took the training samples and their corresponding levels as input. The classifier of the KCF was trained in the Fourier domain using an input image x with M×N pixels that was centered around the target object. The local search size of the target was set to PW×PH, where W×H is the target size and P is an expansion coefficient of the search region. The KCF method considered all the cyclic shifts $x_{m,n},(m,n)\in \{0,\ldots ,M-1\}\times \{0,\ldots ,N-1\}$ as the training samples for the classifier, and their corresponding labels y(m,n) were computed by a Gaussian function. In the KCF tracker, the labels were continuous value from 0 to 1, which takes 1 for the centered target object and smoothly decreases to 0 as the distance increases.

In the KCF tracker, the goal of the training was to solve the linear equation $f(x)=w^{T}x$ that minimizes the cost function, and x represents the extracted features from the input sample. The objective function of the ridge regression problem can be summarized as

(1) $$w=\underset{w}{\min}\sum_{m,n}|\langle \phi (x_{m,n}),w\rangle -{y(m,n)|}^{2}+\lambda {\left\|w\right\|}^{2}$$

where $x_{\left(m,n\right)}$ is the training sample, $y_{\left(m,n\right)}$ is the Gaussian regression target, λ is the regularization term, and 〈⋅,⋅〉 is the inner product.

To introduce the kernel trick for increasing the rich classifier, the input x can be mapped to a nonlinear multidimensional feature space with ϕ(x) and w can be represented as $w=\sum_{i}\alpha_{i}\phi (x_{i})$, which is the linear combination of the learning samples with the coefficient α. The element-wise product of $\phi (x_{i})$ can be computed using the kernel function $k(x,x^{\prime })=\langle \phi (x),\phi (x^{\prime })\rangle$. The objective function can be expressed as

(2) $$f(x)=w^{T}x=\left|\sum_{i}\alpha_{i}\phi^{T}(x_{i})\right|\phi (x)=\sum_{i}\alpha_{i}k(x_{i},x^{\prime })$$

According to the theorem [39], the solution for this regression problem can be computed by

(3) $$\alpha ={\left(K+\lambda I\right)}^{-1}y$$

where K is the kernel matrix and α is the vector of coefficients $\alpha_{i}$. Since, matrix K is circulant, the online classifier coefficient α can be efficiently computed in the Fourier domain as

(4) $$\hat{\alpha }=\frac{\hat{y}}{{\hat{k}}^{xx}+\lambda }$$

where ˆ denotes a Discrete Fourier Fransform (DFT), $k^{xx}$ is the kernel correlation, and y is a m×n label matrix computed by a Gaussian function. The fraction represents element-wise division. In this study, we used the Gaussian kernel. If we compute the kernel k between x and $x^{\prime }$, then the Gaussian kernel $k^{xx^{\prime }}=\exp\left(-\frac{1}{\sigma^{2}}({\left\|x-x^{\prime }\right\|}^{2})\right)$ can be rewritten in Equation (5) as

(5) $$k^{xx^{\prime }}=\exp\left(-\frac{1}{\sigma^{2}}({\left\|x\right\|}^{2}+{\left\|x^{\prime }\right\|}^{2}-2F^{-1}({\hat{x}}^{*}⊙{\hat{x}}^{\prime }\right)$$

where ⊙ represents the element-wise products and ${\hat{x}}^{*}$ represents the complex conjugate of $\hat{x}$.

During the detection step, we also used the circulant matrix trick to increase the computation speed during the tracking process. In the subsequent frames, the target object position was obtained by the train coefficient vector α and base sample x. If the new patch is z with the same search window size of x, the confidence score of z is computed by

(6) $$R_{Z}=f(z)=F^{-1}({\hat{k}}_{\hat{x}z}⊙\hat{\alpha })$$

where $F^{-1}$ represents the inverse fast Fourier transform, ˆ denote the Fourier transform, $\hat{x}$ represents the learned target appearance model, and $\hat{\alpha }$ represents the learned classifier coefficients. The response scores for all cyclic-shifted cropped images are stored in f(z). The new location of the target in the current frame can be detected by searching the position with the highest response score.

The main differences between our method and KCF trackers are given as follows: (1) KCF and our method both use kernel correlation and circulant structure matrix for object detection and training; (2) KCF uses only HOG features, but our approach uses HOG, CN, and gray intensity features; (3) KCF has no strategy to detect target appearance changes such as occlusion, but our approach has an appearance-change detection strategy based on PSR; (4) KCF updates the model parameters with a fixed learning rate in every frame, but our approach updates the model parameters with a dynamic learning rate only when the target object is reliable; (5) The KCF tracker has no strategy to estimate the scale variations, but our method has a correlation filter-based scale estimation strategy.

### 3.2. Multiple Features Integration

Object features are an important factor for object tracking to discriminate the target from the background. In general, multichannel features contain more representative information than single-channel features in separating the background and foreground. In this study, we integrated three features such as HOG, color names, and gray intensity to form multidimensional features. Suppose we have d feature channels for the image data and these feature channels can be concatenated to form a vector $x=[x_{1},x_{2},x_{3},\ldots ,x_{d}]$. The multichannel kernel correlation can be calculated as

(7) $$k^{xx^{\prime }}=\exp\left(-\frac{1}{\sigma^{2}}({\left\|x\right\|}^{2}+{\left\|x^{\prime }\right\|}^{2}-2F^{-1}(\sum_{d=1}^{d}{{\hat{x}}_{d}}^{*}⊙{{\hat{x}}^{\prime }}_{d}))\right)$$

Equation (7) helps us to combine different features and construct richer multichannel features rather than a single-channel feature. In this study, we used 31-dimensional HOG gradient orientation descriptors [28,40]. We also extracted 10-dimensional color names [19,41] for the color images and 1-dimensional gray features for both color and gray-image sequences. These three features were integrated to improve the training and detection performance.

### 3.3. Online Model Updating and Dynamic Learning Rate Strategies

Correlation-filter-based trackers such as [16,17,18,19,20,21,22] have no strategy to detect object appearance changes and these trackers update their appearance models for each frame with the fixed learning rate. This model’s updated technique performed well when the appearance changes occurred slowly and there was no occlusion. However, this update strategy failed to detect the appropriate target position when the tracker faced some challenging situations, such as illumination variation, deformation, abrupt motion change, and occlusion. Moreover, this tracking model’s updating strategy increased the computational cost. An appropriate model updating strategy is most important to increase the performance of the tracker. We mainly focused on the learning rate and reliable patch to update the target model. To select the reliable target appearance and dynamic learning rate, we computed the peak-to-sidelobe ratio (PSR) of the input image. The PSR function in our tracking method is given as

(8) $$PSR=\frac{\max(f(z))-\mu_{\Phi }(f(z))}{\sigma_{\Phi }(f(z))}$$

where z is an input image patch and the corresponding response map is denoted by f(z). Φ denotes the sidelobe size around the peak. $\sigma_{\Phi }$ and $\mu_{\Phi }$ represents the standard deviation and mean of the sidelobe, respectively. The central region in this study was set as 15% of the response map area.

**Figure 2:**
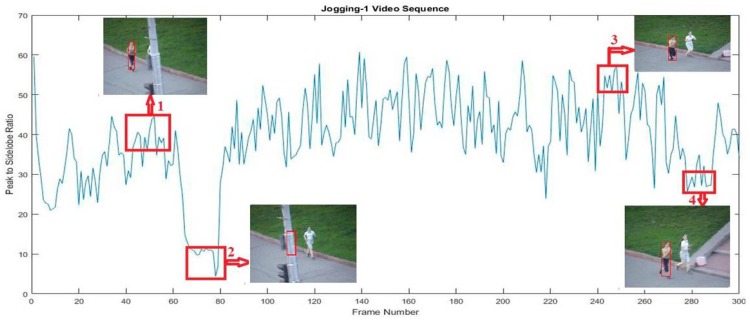
PSR curve analysis using Jogging-1 sequence.

To understand the properties of the PSR curve, we give an example in Figure 2. The x-axis and y-axis represent the frame number and PSR value in each frame, respectively. The red boxes indicate the change regions. From the Figure 2, we can easily observe that the PSR curve dramatically decreased when the target object underwent some appearance variations, such as occlusion, deformation, and other reasons. Furthermore, we also observed that the PSR curve dramatically increased when the target object recovered from the abnormal conditions. Regions 1 and 3 were normal steps. In region 2, the target was occluded by another object. In region 4, the target was partially occluded by the wire and a deformation problem occurs. From this observation, we used a PSR value to select an appropriate target appearance to update the current model. If the PSR value was lower than the predefined threshold value, the current object’s appearance was considered to be corrupted and we stopped the model updating process. The model was updated when the PSR value was larger than the threshold. We also introduced an approach for dynamically determining the learning rate factor $\eta_{f}$, which is defined as the ratio of the PSR value in the current frame to the mean PSR value in the historical frames:

(9) $$\eta_{f}=\frac{P_{t}}{\frac{1}{t-i}\sum_{i}^{t}P_{i}}$$

where $P_{t}$ is the PSR value in the current frame t, i is the initial frame number, and t>i. We compute the learning rate $\eta_{t}$ of the current frame by using the initial learning rate η and the learning-rate factor $\eta_{f}$ as follows:

(10) $$\eta_{t}=\eta *\eta_{f}$$

The classifier coefficient $\hat{\alpha }$ and the target appearance model $\hat{x}$ of the translation filter were updated using Equation (11) when the PSR value increased the level of the threshold value T as follows:

(11) $$\left\{ \begin{array}{l} {\hat{\alpha }}_{t}=(1-\eta_{t}){\hat{\alpha }}_{t-1}+\eta_{t}\alpha \\ {\hat{x}}_{t}=(1-\eta_{t}){\hat{x}}_{t-1}+\eta_{t}x \end{array}\right.$$

where t denotes the present frame, α and x represent the newly computed model, and ${\hat{\alpha }}_{t}$ and ${\hat{x}}_{t}$ represent the current updated model.

### 3.4. Scale Variation Estimations

To handle the scale changes of the target object, we constructed a separate correlation-filter-based scale filter similar to DSST [17], which is shown in Figure 1. Our scale filter was dependent on the translation filter because the input images for the scale filter were cropped from the current frame using the same target location predicted by the translation filter. To generate a scale filter pyramid, we used 33 image samples with the same center location predicted by the translation filter, but the size of the image samples was different and each of the image sample sizes was determined by $A^{s}(W^{t}\times H^{t})$, where W×H denotes the target size, t is the current frame, scale factor is denoted by A, and $s\in \left\{\left\lfloor -\frac{S-1}{2}\right\rfloor ,\ldots ,\left\lfloor \frac{S-1}{2}\right\rfloor \right\}$. Before extracting the features of each sample, the patches were resized with the same template size. We used HOG features for training and detection of the scale filter. The filter responses of the image samples were stored in a one-dimensional array. The current scale factor was estimated by finding the maximum response from the correlation response maps. Let $R_{s}$ represent the correlation response maps of the scale filter, the optimal scale s can be obtained by

(12) $$s=\underset{s}{\arg\max}(R_{s})$$

The scale filter classifier coefficient $\hat{\alpha }$ and the target appearance model $\hat{x}$ were updated by Equation (13) when the maximum scale filter response was greater than the threshold T.

(13) $$\left\{ \begin{array}{l} {\hat{\alpha }}_{t}=(1-\eta ){\hat{\alpha }}_{t-1}+\eta \alpha \\ {\hat{x}}_{t}=(1-\eta ){\hat{x}}_{t-1}+\eta x \end{array}\right.$$

where η represents the scale filter learning rate.

## 4. Results and Discussion

To evaluate the experimental data analysis, we used three challenging object tracking datasets, namely, the OTB-2015 [29], Temple Color 128 [30], and MEEM [31] datasets. The evaluation was measured using three metrics including overlap precision (OP), distance precision (DP), and area under the curve (AUC). Firstly, we present the experimental setup and evaluation metric used in our experiments. Secondly, we provide the quantitative analysis of our proposed method with the other related state-of-the-art trackers on the OTB-2015 and Temple Color 128. Thirdly, we describe the experimental results on MEEM dataset. Fourthly, we present qualitative experimental results on the OTB-2105 datasets. Finally, the attribute-based comparisons of the state-of-the-art trackers and our tracker are described.

### 4.1. Experimental Setup and Evaluation Methodology

We ran our algorithm in MATLAB 2015b with a 64-bit Windows environment. The hardware environment included a PC with Intel Core i9-7900X 3.30 GHz CPU and 32 GB RAM. In our experiments, the initial position of the target was identified by the ground truth in the first frame. The size of the search window for the translation filter was set to 2.2 times the target size of the first frame. The regularization parameter λ was set to $10^{-4}$. The Gaussian kernel bandwidth σ was set to 0.5. The HOG cell size and the number of the HOG orientation bin were set to 4×4 and 9, respectively. The initial model updating rate η was set to 0.02. Similar to DSST [17], we used 33 numbers of scales with a scale factor of 1.02 and a scale-learning rate of 0.025 in the scale model. Finally, the extracted features for each filter were always multiplied by a Hann window. All parameters were the same for all videos in the benchmarks.

We used three object-tracking datasets to implement our proposed method, namely, OTB-2015, Temple Color 128, and MEEM. The OTB-2015 dataset contains 100 video sequences. All these video sequences are manually annotated with 11 different attributes which cover various challenging problems, including background clutters (BC), deformation (DEF), fast motion (FM), illumination variation (IV), in-plane rotation (IPR), low resolution (LR), motion blur (MB), occlusion (OCC), out-of plane rotation (OPR), out-of-view (OV), and scale variation (SV). The Temple Color 128 dataset has 128 challenging color video sequences. To evaluate the effectiveness of our occlusion mechanism, we also used the MEEM dataset that contains 5 heavy occlusion video sequences out of 10.

To assess the tracking performance and the display of the experimental results, we used one-pass evaluation (OPE) protocol as suggested in [29]. In this protocol, the methods run throughout the video sequence from the first frame and display the average distance precision (DP) and overlap precision (OP) rate. The distance precision (DP) is calculated as the percentage of frames in a sequence, where the Euclidean distance between the tracker output and the ground-truth center positions of the target is lower than a certain threshold. The overlap precision (OP) is calculated as the percentage of frames in a sequence where the intersection and union between the tracked and ground-truth bounding boxes are higher than a certain threshold. In the success plot, the trackers are ranked by the area under the curve (AUC), which is the average success scores corresponding to the sampled overlap thresholds.

### 4.2. Quantitative Analysis of OTB-2015

We compared our proposed method with 13 state-of-the-art methods such as tracking learning detection (TLD) [42], compressive tracking (CT) [43], distribution fields tracking (DFT) [44], locally orderless tracking (LOT) [45], CSK [16], CN [19], tracking with Gaussian process regression (TGPR) [46], DSST [17], KCF [18], fDSST [36], SAMF [20], MUSTer [38] and LCT [37].

**Figure 3:**
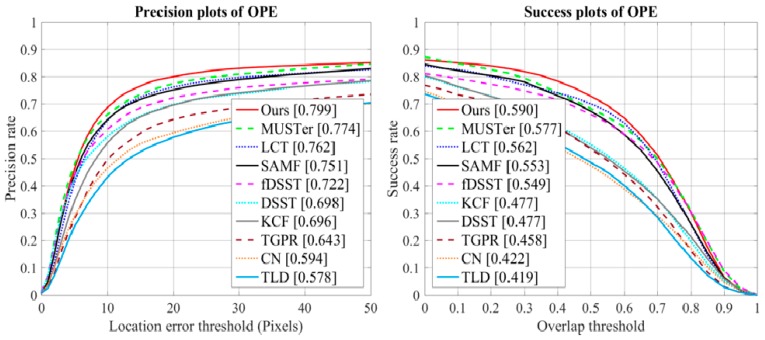
Precision and success plots on OTB-2015 using OPE.

The experimental results of the precision and success plots on the OTB-2105 dataset are shown in Figure 3. Among the compared trackers in the literature, the MUSTer tracker provides the second-best results on the OTB-2015, with a mean DP of 77.4% and an AUC of 57.7%. Our method showed the best tracking performance, with a mean DP of 79.9% and a mean AUC of 59.0% on OTB-2015 dataset compared to the other trackers. The LCT tracker based on KCF and an online fern classifier obtained a precision score of 76.2% and an AUC score of 56.2%. The SAMF tracker extended the KCF tracker with scale estimation and multiple feature integration, obtaining a distance precision score of 75.1% and an AUC score of 55.3%. Compared to the SAMF tracker, our approach outperformed it by 4.8% and 3.7% in average DP and AUC, respectively. Compared to the baseline KCF tracker, the mean DP and AUC scores of our proposed method improved by 10.3% and 11.3%, respectively.

Table 1 shows a comparison between our method and others on the OTB-2015 dataset using mean overlap precision (OP) and tracking speed. Our method obtains a mean OP score of 72.9% and outperforms LCT, MUSTer, and SAMF by 2.8%, 4.6% and 5.5%, respectively. Compared with the tracking speed, our method runs at 74 frames per second (FPS) and provides the second-best results.

### 4.3. Robustness to Initialization Analysis of OTB-2015

**Figure 4:**
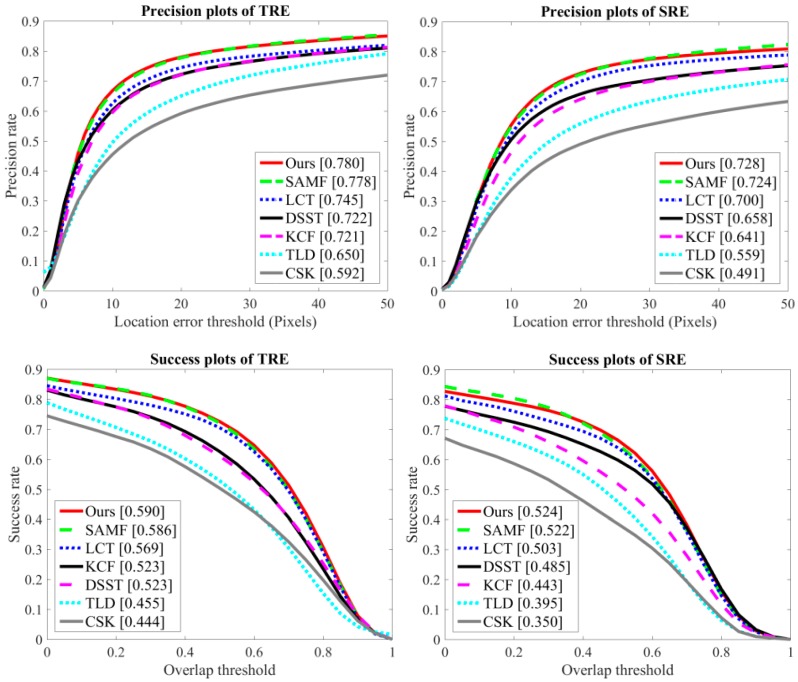
Precision and success plots on OTB-2015 using TRE and SRE.

To assess the robustness of our approach, we used spatial robustness evaluation (SRE) and temporal robustness evaluation (TRE) criteria as discussed in [29]. In the TRE, the tracker is evaluated twenty times from the different starting frames with the corresponding ground-truth bounding box position until the end of the video sequence. In this case, each image sequence is divided into 20 segments. The TRE score is generated by the average of these 20 tests. In the TRE, the tracker is evaluated by initializing the bounding box at 12 different locations using shifting and scaling of the ground-truth location in the first frame. In this case, the trackers execute each video sequence with 12 different initializations. The SRE score is calculated by the average of these 12 executions. Figure 4 shows the precision and success curves for TRE and SRE evaluations on the OTB-2015 dataset with 100 video sequences. We included the seven trackers in Figure 3 for robustness evaluation. Among the existing approaches, SAMF and LCT provide the best results. In these evaluations, our approach obtained the top rank over these trackers.

### 4.4. Quantitative Analysis of Temple Color 128

**Figure 5:**
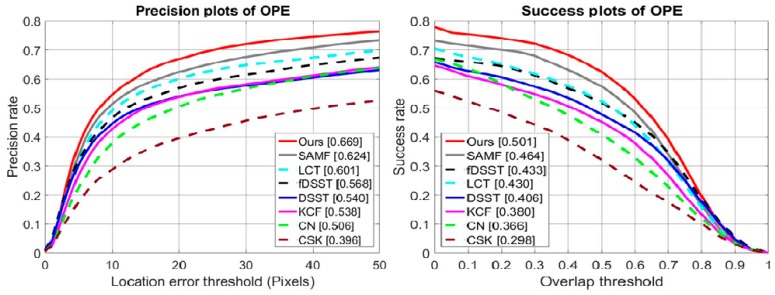
Precision and success plots on Temple Color-128.

To evaluate the effectiveness of our approach, we used the Temple Color 128 dataset and performed comprehensive experiments on this dataset. The Temple Color 128 dataset has 128 color video sequences. We compared our method with seven well-known tracking methods (CSK, KCF, fDSST, LCT, SAMF, CN, and DSST). Figure 5 shows the comparison of the results based on DP and AUC scores. In this dataset, the SAMF tracker performed better than the LCT tracker in both precision and success plots due to the integration of color-naming features. In the precision and success curve, the proposed method achieved the best performance on the Temple Color 128 dataset with DP of 66.9% and AUC of 50.1%. The integration of multiple features, the appearance-change detection mechanism, and the online model updating technique improved overall performance on this dataset. Compared to the SAMF [20], our approach improved the precision score and success score by 4.5% and 3.7%, respectively. Compared to the LCT tracker [37], our method increased DP and AUC scores by 6.8% and 7.1%, respectively. The precision and success scores of our method were especially improved, with large a margin compared to the base tracker KCF [18] by 13.1% and 12.1%, respectively.

### 4.5. Results Analysis of the MEEM Dataset

The correlation-filter-based tracers such as [16,17,18,19,20,21,22] have no strategy to detect target appearance changes and occlusions. To analyze the occlusions and appearance-change effectiveness, we used the MEEM dataset [31]. The MEEM dataset has 10 challenging video sequences with more than 7500 frames. The sequences were collected from the real-world videos that reflect occlusions and appearance changes.

**Figure 6:**
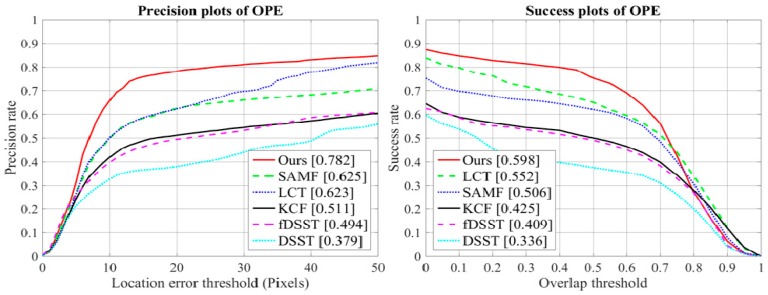
Precision and success plots of the MEEM dataset.

We used OTB evaluation metrics to draw the precision curve and success curve. The experimental results of our method and other methods are shown in Figure 6. In this dataset, our occlusion and appearance-change detection step properly handled these problems and achieved the best tracking performance. The LCT tracker provided the second-best results based on the success rate due to the kernel version of the correlation filters and the redetection module when tracking failure. Ours occlusion-handling mechanism can properly detect partial and full occlusion and can stop the model from updating until the target object reappears. Our proposed method achieved the best results with an average DP of 78.2% and AUC of 59.8%. Compared to the baseline KCF tracker, our precision rate and success rate have been increased by 27.1% and 17.3%, respectively. Compared to the LCT tracker, our precision rate and success rate have been increased by 15.9% and 4.6%, respectively.

**Figure 7:**
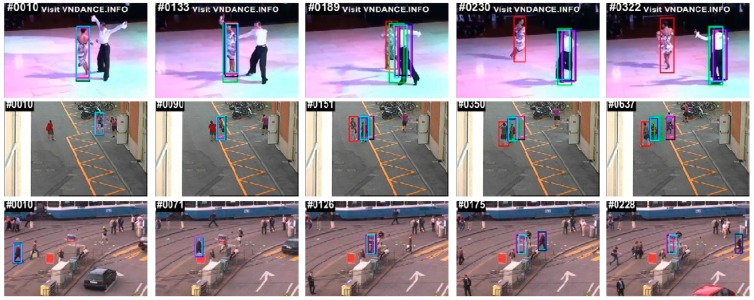
Qualitative analysis of three challenging sequences (Latin, Ped2 and Ped1) using the MEEM dataset. Ours, SAMF, LCT, fDSST, **KCF** and DSST.

The qualitative results of some selective sequences are shown in Figure 7. In the Ped2 and Latin sequences, all of the trackers failed to recover the target except our method when the target object underwent full occlusion. In the Ped1 sequence, the KCF, DSST, and fDSST lost the target when the target object underwent partial occlusion, but LCT, SAMF, and our methods detected the target with a low center location error (CLE).

### 4.6. Qualitative Analysis of OTB-2015

**Figure 8:**
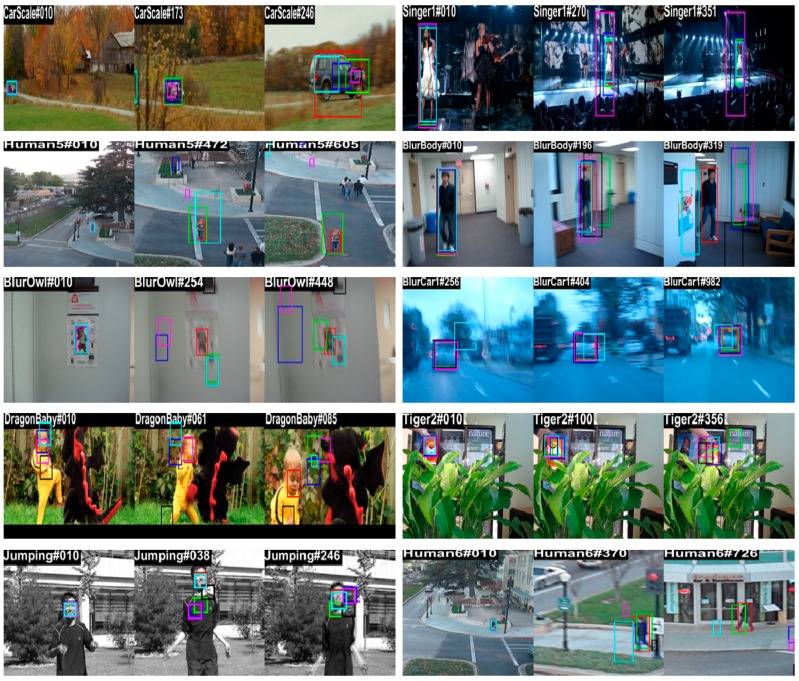
Qualitative results of six trackers: Ours, MUSTer, SAMF, DSST, **KCF** and TLD.

**Figure 9:**
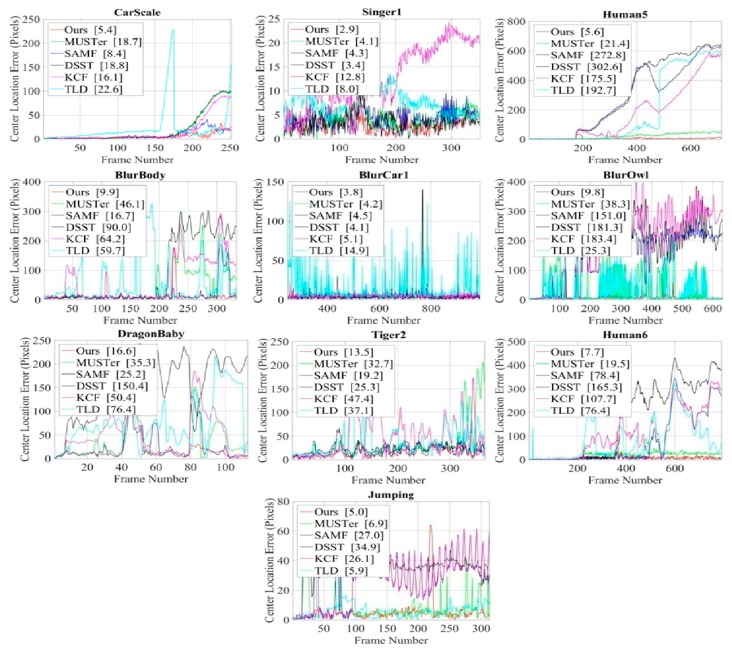
Center location error results for 10 challenging sequences.

Figure 8 shows the qualitative analysis of our method compared with the existing five methods (MUSTer, SAMF, DSST, KCF, and TLD) for different key frames of 10 representative challenging sequences and the results are displayed by the tracking bounding boxes. The center location error results corresponding to these sequences are shown in Figure 9. The center location errors (CLE) are calculated as the Euclidean distance between the trackers estimated location and the ground-truth location of the target.

The Carscale, Singer1, and Human5 sequences have large scale-variation problems. At the beginning of the Carscale and Human5 sequences, the target object is small, but over time, the target size increases dramatically, and at the end of these sequences, the object appears large. At the beginning of the Singer1 video, the target size is large and decreases dramatically over time. Thus, it is very challenging to detect and estimate the target-scale state properly. The MUSTer, SAMF, and DSST trackers used a scale-change estimation strategy, but they failed to locate the accurate scale changes. However, our proposed scale filter and adaptive scale model updating strategy could estimate scale variations accurately and provide the lowest CLE value. In the Singer1 sequence, MUSTer, SAMF, and DSST trackers located the target center properly. However, our method was able to locate the center position and scale variation accurately with a low center-error rate.

Motion blur is another problem that occurs due to fast object movement and camera shaking. The BlurBody, Blucar1, Jumping, and BlurOwl sequences have motion blur and fast motion problems. Most of the trackers could not track the target position in these challenges due to the small search area and the linear model updating strategy. Our object search area and restricted model updating strategies could handle these problems with the lowest CLE value.

DragonBaby and Tiger2 are complex video sequences with deformation, occlusion, and fast motion. Most of the trackers failed to detect the actual target position when the target movement between two consecutive frames was higher, when there was object deformation, and when it was partially occluded by some other object. Only our method tracked the target position with a small center location error rate. Another challenging sequence called Human6 has large scale changes and full occlusion. The MUSTer tracker had an occlusion and scale-handling strategy, but it also failed detect the target position. Only our scale and occlusion-handling mechanism could detect the target state properly.

### 4.7. Attribute-Based Analysis of OTB-2015

The attribute-based results analysis of our method with other state-of-the-art methods on the OTB-2015 dataset is shown in Table 2, Table 3 and Table 4.

Table 2 shows the distance precision scores at a threshold of 20 pixels on this dataset under different attributes. The proposed method performs well against the state-of-the-art trackers when evaluating 11 challenging aspects. Among the 11 attributes, our method ranked best in 7 attributes, second in IPR attributes, and third in IV, BC, and LR. The second-best tracker, MUSTer, obtained the best results in IV and BC and second best in SV, OCC, DEF, and LR. The LCT tracker had the best result in IPR. In the scale variation challenge, our method achieved an improvement of 11.8%, 8.5%, 8.9%, 7.0%, 4.1% and 4.6% compared to the KCF, DSST, fDSST, LCT, MUSTer, and SAMF, respectively. In the occlusion attribute, our results improved by 15.1%, 9.9%, 4.7% and 5.5% compared to the KCF, LCT, MUSTer, and SAMF, respectively. This is mainly because we use scale filter predicted outputs at the time of translation filter update and adaptively update the scale filter. In terms of fast motion, our method provided gains of 12.9%, 6.0%, 6.9%, 6.7% and 9.6% compared to the KCF, fDSST, LCT, MUSTer, and SAMF, respectively. In the case of motion blur, our results improved by 15.6%, 7.7%, 8.8%, 7.9% and 10.2% compared to the KCF, fDSST, LCT, MUSTer, and SAMF respectively. These improvements are mainly due to the object padding size and online model updating strategy, which stop the model updating step when an abnormal PSR score is detected.

Table 3 presents the AUC scores for the different challenging attributes in the same dataset. Our method achieved the best results in 8 attributes out of the 11. On the BC and LR attributes, the fDSST method achieved the best result with AUC scores of 0.585 and 0.429, while our method obtained AUC scores of 0.537and 0.399. In the IV attribute, MUSTer provided the highest score with AUC of 0.600, whereas our AUC score was 0.558.

Table 4 presents the success rate at a threshold of 0.5 for the OTB-2015 dataset. In this case, our method achieved the best results for seven attributes, LCT had the best results for two attributes, and fDSST had the best results for two other attributes.

## 5. Conclusions

In this study, we presented new appearance-variation detection and online model updating strategies based on the signal strength of PSR. We improved the KCF tracker by adding scale adaptation, occlusion detection, and online model updating techniques. We integrated multiple features such as HOG, color names, and gray intensity with the KCF translation filter to improve the overall performance. The scale adaptation based on correlation filters and an adaptive scale model updating strategy can accurately estimate the target scale. Based on our proposed PSR curve property, we restricted the linear model updating strategy and only updated the model with the reliable target object. We also introduced an adaptive model learning rate strategy that automatically adjusted the learning rate. Our scale adaptation, occlusion handling, and online model updating techniques can easily be integrated into any other tracking framework. To assess the effectiveness of our proposed method, we conducted experiments on the three datasets. The experimental outputs show that our method performs well against the other methods in terms of accuracy, robustness, and efficiency. Moreover, our method is more powerful in addressing the problems of occlusion, scale variation, fast motion, and motion blur and can run at a high speed.

## Figures and Tables

**Table 1:** Mean overlap precision (OP) scores and tracking speed on the OTB-2015. The first, second and third highest results are colored by red, green and blue.

	TLD	TGPR	DSST	KCF	fDSST	SAMF	MUSTer	LCT	Ours
Mean OP (%)	48.6	53.5	54.0	55.1	66.2	67.4	68.3	70.1	72.9
Speed (FPS)	21	1	41	113	59	17	2	21	74

**Table 2:** Attribute-based analysis of OTB-2015 (mean DP score). The first, second and third highest results are colored red, green and blue, respectively.

Attribute	CSK	DSST	fDSST	KCF	LCT	MUSTer	SAMF	TGPR	TLD	Ours
IV(38)	0.482	0.730	0.746	0.719	0.746	0.782	0.715	0.633	0.549	0.734
OPR(63)	0.489	0.670	0.666	0.677	0.746	0.744	0.739	0.642	0.549	0.771
SV(64)	0.448	0.666	0.662	0.633	0.681	0.710	0.705	0.599	0.549	0.751
OCC(49)	0.428	0.620	0.640	0.630	0.682	0.734	0.726	0.594	0.498	0.781
DEF(44)	0.451	0.574	0.611	0.617	0.689	0.689	0.686	0.630	0.454	0.729
MB(29)	0.355	0.611	0.680	0.601	0.669	0.678	0.655	0.529	0.527	0.757
FM(39)	0.397	0.584	0.690	0.621	0.681	0.683	0.654	0.533	0.538	0.750
IPR(51)	0.514	0.724	0.727	0.701	0.782	0.773	0.721	0.659	0.590	0.777
OV(14)	0.276	0.487	0.578	0.501	0.592	0.591	0.628	0.493	0.459	0.708
BC(31)	0.574	0.702	0.779	0.713	0.734	0.784	0.689	0.593	0.465	0.735
LR(9)	0.445	0.708	0.675	0.671	0.699	0.747	0.766	0.622	0.625	0.743

**Table 3:** Attribute-based analysis of OTB-2015 (success rate based on AUC score). The first, second and third highest results are colored red, green and blue, respectively.

Attribute	CSK	DSST	fDSST	KCF	LCT	MUSTer	SAMF	TGPR	TLD	Ours
IV(38)	0.368	0.494	0.563	0.479	0.566	0.600	0.534	0.452	0.407	0.558
OPR(63)	0.354	0.448	0.499	0.453	0.538	0.537	0.536	0.455	0.380	0.564
SV(64)	0.318	0.412	0.497	0.394	0.488	0.512	0.495	0.404	0.385	0.543
OCC(49)	0.331	0.430	0.484	0.443	0.507	0.554	0.540	0.429	0.353	0.584
DEF(44)	0.337	0.417	0.469	0.436	0.499	0.524	0.509	0.455	0.329	0.534
MB(29)	0.308	0.467	0.536	0.459	0.533	0.544	0.525	0.429	0.432	0.602
FM(39)	0.329	0.442	0.547	0.459	0.534	0.533	0.507	0.420	0.424	0.575
IPR(51)	0.379	0.485	0.541	0.469	0.557	0.551	0.519	0.462	0.417	0.559
OV(14)	0.250	0.374	0.457	0.393	0.452	0.469	0.480	0.373	0.356	0.554
BC(31)	0.410	0.477	0.585	0.498	0.550	0.581	0.525	0.428	0.361	0.537
LR(9)	0.234	0.314	0.429	0.290	0.399	0.415	0.425	0.344	0.350	0.399

**Table 4:** Attribute-based analysis of OTB-2015 (mean OP score). The first, second and third highest results are colored red, green and blue, respectively.

Attribute	CSK	DSST	fDSST	KCF	LCT	MUSTer	SAMF	TGPR	TLD	Ours
IV(38)	0.393	0.558	0.690	0.549	0.717	0.714	0.647	0.533	0.469	0.683
OPR(63)	0.378	0.509	0.592	0.527	0.676	0.636	0.660	0.543	0.424	0.697
SV(64)	0.307	0.437	0.577	0.416	0.586	0.584	0.590	0.450	0.425	0.655
OCC(49)	0.349	0.482	0.585	0.512	0.635	0.659	0.669	0.517	0.398	0.740
DEF(44)	0.353	0.462	0.560	0.503	0.620	0.635	0.613	0.542	0.372	0.669
MB(29)	0.345	0.553	0.651	0.550	0.659	0.652	0.641	0.511	0.507	0.764
FM(39)	0.368	0.498	0.659	0.526	0.655	0.622	0.595	0.471	0.492	0.714
IPR(51)	0.413	0.566	0.652	0.553	0.694	0.648	0.641	0.555	0.453	0.679
OV(14)	0.274	0.421	0.548	0.457	0.531	0.541	0.551	0.452	0.371	0.682
BC(31)	0.465	0.563	0.713	0.609	0.703	0.683	0.639	0.543	0.406	0.669
LR(9)	0.222	0.274	0.561	0.253	0.484	0.442	0.495	0.381	0.395	0.432
